# Reactive oxygen species and male reproductive hormones

**DOI:** 10.1186/s12958-018-0406-2

**Published:** 2018-09-11

**Authors:** Mahsa Darbandi, Sara Darbandi, Ashok Agarwal, Pallav Sengupta, Damayanthi Durairajanayagam, Ralf Henkel, Mohammad Reza Sadeghi

**Affiliations:** 1 Reproductive Biotechnology Research Center, Avicenna Research Institute, Academic Center for Education, Culture and Research, Tehran, Iran; 2 American Center for Reproductive Medicine, Cleveland Clinic, Cleveland, Ohio 44195 USA; 3 Department of Physiology, Faculty of Medicine, MAHSA University, Jalan SP2, Bandar Saujana Putra, 42610 Jenjarom, Selangor Malaysia; 4 Department of Physiology, Faculty of Medicine, Universiti Teknologi MARA, Sungai Buloh Campus, Jalan Hospital, 47000 Sungai Buloh, Selangor Malaysia; 5 Department of Medical Biosciences, University of the Western Cape, Bellville, Cape Town, 7535 South Africa; 6 Reproductive Immunology Research Center, Avicenna Research Institute, Academic Center for Education, Culture and Research, Tehran, Iran

**Keywords:** Antioxidants, Hypothalamic-pituitary-gonadal axis, Male infertility, Oxidative stress, Reactive oxygen species, Testosterone

## Abstract

Reports of the increasing incidence of male infertility paired with decreasing semen quality have triggered studies on the effects of lifestyle and environmental factors on the male reproductive potential. There are numerous exogenous and endogenous factors that are able to induce excessive production of reactive oxygen species (ROS) beyond that of cellular antioxidant capacity, thus causing oxidative stress. In turn, oxidative stress negatively affects male reproductive functions and may induce infertility either directly or indirectly by affecting the hypothalamus-pituitary-gonadal (HPG) axis and/or disrupting its crosstalk with other hormonal axes. This review discusses the important exogenous and endogenous factors leading to the generation of ROS in different parts of the male reproductive tract. It also highlights the negative impact of oxidative stress on the regulation and cross-talk between the reproductive hormones. It further describes the mechanism of ROS-induced derangement of male reproductive hormonal profiles that could ultimately lead to male infertility. An understanding of the disruptive effects of ROS on male reproductive hormones would encourage further investigations directed towards the prevention of ROS-mediated hormonal imbalances, which in turn could help in the management of male infertility.

## Background

Over the past 40 years, reports regarding the decline in semen quality [1–4] and its probable consequences on male fertility have encouraged studies about the effects of environment and lifestyle factors on the male reproductive potential. Reactive oxygen species (ROS) produced by exogenous and endogenous factors are highly reactive oxygen derivatives with half-lives in the nano- to milliseconds range. These molecules reportedly play a key role in altering male reproductive functions [5, 6]. Lifestyle modifications, technological advancements, escalating levels of pollution, alcohol consumption, smoking of cigarettes and vaping, and physical stress are among the prime exogenous causes of ROS production [7–9]. Also, multiple mechanisms involving metabolism in the cell membrane, mitochondria, peroxisomes, and endoplasmic reticulum can produce endogenous ROS [7, 9].

Antioxidants defend against excessive ROS levels through enzymatic (superoxide dismutase, catalases, and peroxidases) and non-enzymatic (vitamins, steroids etc.) mechanisms [7, 10]. In cases where the imbalance between oxidants (ROS) and antioxidants leans towards the oxidants, oxidative stress (OS) occurs, which puts the cells and the body under stress. As a result, excessive ROS can induce lipid peroxidation, disrupt DNA, RNA as well as protein functions in the spermatozoa and other testicular cells [10].

High ROS levels can increase the possibility of infertility not only directly by inducing OS, but also indirectly by acting through the hypothalamic axes of hormone release [11–13]. ROS reduce male sex hormone levels and disrupt the hormonal balance that regulates male reproductive functions [14], and thus causes infertility. These “endocrine disruptors” not only interfere in the communication between testis and the hypothalamic-pituitary unit, they also disrupt the cross-talk between the hypothalamic-pituitary-gonadal (HPG) axis with other hypothalamic hormonal axes [15, 16]. The testis, as the primary male sex organ, is not only concerned with spermatogenesis, but also with the secretion of several hormones [17] which are required for regulation of gonadotropin secretion, spermatogenesis, formation of male phenotype during sexual differentiation, and normal sexual behaviour [18]. Hence, by interfering with normal hormonal release, ROS disrupt these essential reproductive functions.

Therefore, this review precisely elucidates (a) the role of ROS, generated by various exogenous and endogenous factors, in disrupting hormone secretion by interfering in the endocrine pathways, as well as in their cross-talk, (b) hormonal regulation of the oxidative status of male reproduction, and (c) a possible mechanism of action of ROS-induced disruption of the male reproductive hormonal profile.

## Endocrinology of male fertility

The gonadotropin releasing hormone (GnRH) secreted by the hypothalamus regulates the release and secretion of gonadotropins, luteinizing hormone (LH) and follicle-stimulating hormone (FSH) from anterior pituitary that in turn regulate testicular functions [17]. These gonadal steroids as well as the pituitary gonadotropins, via feedback regulatory mechanisms, further establish physiological homeostasis and maintains normal reproductive functions [14, 17, 19]. FSH receptors are located on the membrane of Sertoli cells, while those of LH are on the Leydig cells. They coordinate to synthesize testosterone, maintain normal spermatogenesis, sperm health and density [19–21].

**Fig. 1:**
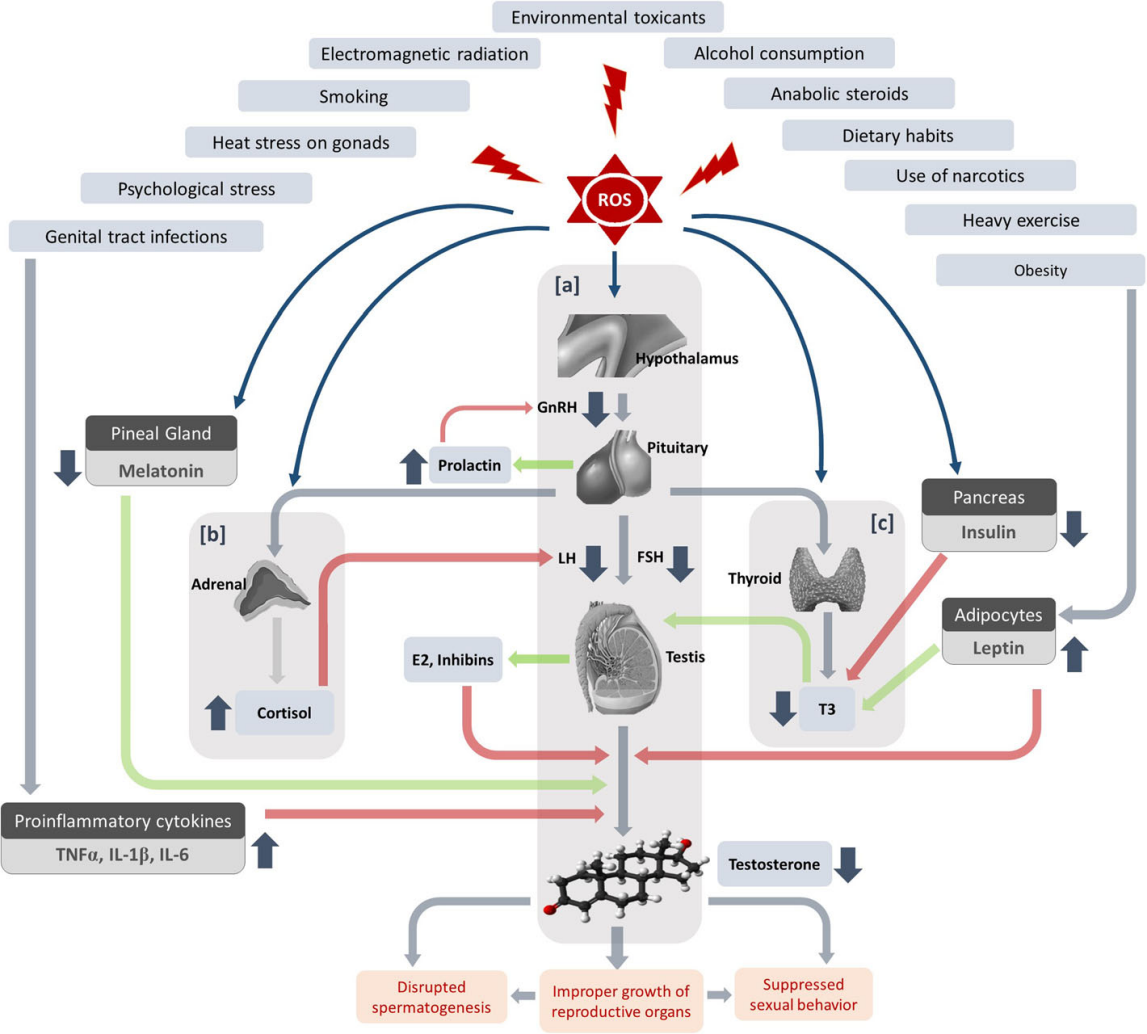
Sources of reactive oxygen species (ROS) and their impact on the complex endocrine network regulating male reproduction. **a** High levels of ROS impact upon the HPG axis which results in decreased secretion of male reproductive hormones. **b** Through the HPA axis, ROS increases the release of the stress hormone cortisol, which through the HPA-HPG axes cross-talk, further decreases LH secretion. **c** Elevated ROS also affects the HPT axis which results in decreased T3 production from the thyroid gland, which through the cross-talk between HPT and HPG axes, again decreases testosterone synthesis. ROS also affects the other endocrine glands which interfere with these endocrine axes to result in decreased testosterone production. Increased oxidative stress (OS), in different conditions, decreases insulin production from the pancreas which again reduces T3 production from the thyroid gland and through HPT-HPG axes cross-talk decreases testosterone biosynthesis. ROS production in obesity also increases circulating leptin levels which directly reduces testosterone synthesis in the testis. Reduced melatonin in OS, and increased production of pro-inflammatory cytokines during reproductive tract infections, affects the HPG axis to reduce testosterone biosynthesis. OS also increases prolactin secretion from the anterior pituitary and E2 synthesis from the testis. These two hormones reduce GnRH secretion from the hypothalamus and testosterone biosynthesis from the testis, respectively

Moreover, other hormones like estradiol (E2) and prolactin (PRL) also take part in the management of male reproductive function. E2, produced both by the testis and via the peripheral conversion of androgenic precursors, is a potent inhibitor of LH and FSH [18, 19] (Fig. 1). PRL-inhibiting GnRH secretion via modulation of dopaminergic pathway may also reduce LH and testosterone level and thus is associated with hypogonadism [22]. Dehydroepiandrosterone (DHEA) is another male reproduction ameliorating, steroid hormone secreted by the adrenal cortex [23, 24]. Inhibin A and B, dimeric hormones produced by Sertoli cells, exhibit negative feedback on FSH secretion and thus also on testicular functions [25]. Moreover, melatonin (MLT), a tryptophan-derived hormone of the pineal gland, positively regulates gonadotropin and testosterone secretion, and thus aid male reproductive functions [26, 27]. Anti-Mullerian hormone (AMH), a dimeric glycoprotein hormone produced in embryonic Sertoli cells, is structurally related to inhibin and is responsible for regression of Mullerian ducts during the first 8 weeks of embryogenesis. It reflects Sertoli cell functions and is inhibited by testosterone under the influence of LH [28–30]. Interactions between the hypothalamo-pituitary-thyroid (HPT) and HPG axes potentially influence testicular development, mostly by the participation of thyroid hormones and FSH [31].

Thus, besides the central control through the HPG axis, the major male reproductive hormones act either individually or via the cross-talks among different endocrine axes to influence male reproductive functions. Consequently, any disruption to these networks may adversely affect male fertility.

## Generation of ROS in the male reproductive tract

Reactive oxygen species (ROS), which are short-lived, unstable, and highly reactive species containing at least one oxygen atom, are able to snatch electrons from other molecules to achieve an electronically-stable state. In this process, the other molecule loses an electron following which a new radical is formed. Subsequently, this radical reacts with another neighbouring molecule, thus passing on the radical status via a reaction called ‘radical-chain reaction’ until two radicals react with one another forming a stable bond. These reactions amplify the degree of alterations in the cellular structures [32–34].

Human spermatozoa contain abundant mitochondria, particularly in its midpiece [35]. An NADH-dependent oxidoreductase (in the inner mitochondrial membrane) and NAD (P) H-oxidase (in the plasma membrane) are two main sources of superoxide (O_2_^●-^) [32, 33, 36]. The majority of ROS generated in human spermatozoa is O_2_^●-^ which is a product of oxidative phosphorylation by addition of an electron to intracellular oxygen and is created between complex I and III of the electron transport chain [37]. H_2_O_2_ is an uncharged, membrane permeable molecule which has been found to be the major initiator of peroxidative damage of the plasma membranes of germ cells [34]. In the presence of transition metals, such as iron (Fe^3+^) and copper, O_2_^●-^ and H_2_O_2_ can generate the extremely reactive OH^•^ through the Haber-Weiss reaction, which consist of a reduction of ferric (Fe^3+^) to ferrous ion (Fe^2+^) [38]. In a subsequent second step, called Fenton reaction, Fe^2+^ is oxidized by H_2_O_2_ to Fe^3+^ whereby hydroxide (OH^−^) and the most reactive hydroxyl radical (OH^•^) are formed. Furthermore, O_2_^●-^ has the ability to interact with nitric oxide (NO) to form peroxynitrite (ONOO^−^), subsequent reactions of which may lead to either apoptotic or necrotic cell death [39]. In the male reproductive tract, ROS finally can be generated by one of these sources according to the above-mentioned mechanisms.

In order to produce the immense amount of energy needed for motility, spermatozoa possess numerous mitochondria in the mid-piece of the flagellum. In the mitochondria, disruption of the membrane potential leads to electron leakage in the electron transfer chain and subsequently produces ROS. The Ca^2+^-dependent NADPH oxidase, called NOX5 (encoded by the *NOX5* gene) was initially detected in the human testis, but was also found to be present in the acrosomal and mid-piece regions of human spermatozoa [40]. NOX5 is a major generator of ROS and could subsequently induce OS. This enzyme is activated when Ca^2+^ binds to its cytosolic N-terminal EF-hand and causes conformational changes to the cell through OS [41]. Moreover, during spermatogenesis, the developing spermatozoa extrude their cytoplasm. When spermiogenesis is disrupted and/or excess cytoplasm is not completely extruded (excess residual cytoplasm), the excess cytoplasm will be retained around the mid-piece. Since cytoplasm contains the enzymatic machinery to produce ROS, any hindrance in the elimination of excess cytoplasm would trigger the production of intrinsic amounts of ROS in excess, which, in turn, would lead to oxidative damage of the plasma membrane and sperm DNA [42].

The prostate and seminal vesicles are the major sources of peroxidase-positive leukocytes (polymorphonuclear leukocytes (50 ∼ 60%) as well as macrophages (20 ∼ 30%)) [43, 44]. Inflammatory responses trigger these cells to generate ROS about 100-times more than it is produced under normal conditions [34, 45, 46]. This elevated ROS production is a part of the natural defense mechanisms of these cells, whereby NADPH-production through the hexose monophosphate shunt is elevated. Leukocyte participation in inflammation is closely connected with the accompanying leukocytospermia [47], a condition defined by the World Health Organization (WHO) as semen samples containing more than one million peroxidase-positive leukocytes per milliliter of semen [48]. Varicocele, a condition caused by an abnormal dilation of veins in the pampiniform plexus surrounding the spermatic cord [49], is also associated with elevated levels of seminal ROS [50].

## ROS and male reproductive hormones

ROS generation, which can be elicited through various exogenous and endogenous pathways, may adversely affect the male reproductive potential by interfering with the endocrine axes both individually and via their cross-talks (Table 1).

**Table 1:** Sources of reactive oxygen species (ROS), their mechanism of generation and effects on male reproductive hormones

Sources of ROS	Mechanism of ROS generation	Effects on male reproductive hormones
Exogenous sources		
Psychological stress	By increasing stress hormone (cortisol) levels and activating the immune–inflammatory system	Decreases serum testosterone and LH levels by suppressing androgen synthesis and inducing Leydig cells apoptosis
Heat stress	By decreasing antioxidant enzyme activities, increasing NADPH oxidase activity and disrupting mitochondrial homeostasis	Disrupts Sertoli cell functions, decreases testosterone and LH levels
Environmental toxicants	By activating inflammatory mechanisms and cellular death	Decreases Leydig and Sertoli cell functions, hormonal biosynthesis
Electromagnetic radiations	By decreasing total antioxidant capacity	Decreases serum testosterone and LH levels
Long-term heavy exercise	By stimulating mitochondrial enzymes including NOX and XO	Decreases LH, FSH, and testosterone levels
Obesity	By increasing leptin levels in human endothelial cells and increasing mitochondrial fatty acid oxidation	Activation of the HPG axis stimulates FSH and LH release. Leptin can directly affect the gonads due to its receptor isoforms in gonadal tissue and stimulate steroid secretion, through increasing the GnRH
High-fat and high-protein food	By decreasing natural food antioxidants and free radical scavengers	Decreases testosterone biosynthesis, LH secretion and androgen profile
Alcohol	By stimulating cytochrome P450s enzyme activities in the liver, altering levels of necessary metals in the body, and reducing antioxidant levels	Increases Sertoli cells and Leydig cells apoptosis, reduces serum testosterone, LH and FSH levels
Marijuana and narcotic drugs	By increasing inflammation and cytochrome p53-induced apoptotic cell death	Inhibits GnRH release and LH production, inhibits HPG axis, reduces testosterone level, and increases SHBG level
Smoking	By decreasing oxygen delivery to the testis and the high metabolic requirements of spermatogenesis, releasing a large number of mutagens and metabolites, weakening of the antioxidant defense systems. Stimulation of NOX enzymes	Alters plasma levels of testosterone, prolactin, estradiol, FSH, LH and SHBG by affecting the Leydig and Sertoli cells
Anabolic steroids	By stimulating mitochondrial respiratory chain complexes, inflammatory cytokine release and cellular apoptosis	Disrupts Leydig cell functions, suppresses HPG axis, reduces LH release and thus testicular testosterone biosynthesis
Endogenous sources		
Aging	By decreasing the activities of antioxidant enzymes, alteration in the mitochondrial membrane potential	Increases lipid peroxidation of Leydig cells, LH sensitivity by diminishing LH receptors, reduces the rate of steroidogenesis, testosterone biosynthesis and secretion
Infections of the reproductive tract	Bacterial strains that colonize the male reproductive tract causes inflammatory damage by inducing leukocyte migration, release of cytokines and other inflammatory mediators, activation of macrophages, lymphocytes and other immunoreactive cells	Reduces serum testosterone levels by disrupting the hormonal axis, increase in LH and FSH levels

### Exogenous factors

#### Psychological stress

Psychological stress has been demonstrated as a cause of idiopathic male infertility and several studies have described a correlation between stress and impaired semen parameters [51–53]. It was reported that psychological stress can increase the circulating levels of cortisol and norepinephrine [54]. These hormones have a significant impact on increasing intracellular levels of ROS/reactive nitrogen species (RNS) to have damaging effects on cellular microstructures and activation of the immune and inflammatory systems [54, 55]. Psychological stress inhibits male reproductive functions by directly affecting the action of glucocorticoids on Leydig cells [11]. As a result, circulating testosterone levels decrease through suppression of androgen synthesis and induction of apoptosis of Leydig cells [56]. Psychological stress can also increase the serum levels of corticosterone (in animals) and cortisol (in humans), which then enhance the apoptotic frequency of Leydig cells [57]. Furthermore, during glucocorticoid production by 11β-hydroxysteroid dehydrogenase-1 (11βHSD-1), NADPH was produced as a cofactor that is used for the biosynthesis of steroidogenic enzymes and testosterone [58].

Stress adversely affects steroidogenesis, since changes in the autonomic catecholaminergic activities during stress may suppress Leydig cell functions, thus inhibiting steroidogenic enzyme activities and testosterone production [11]. Stress-induced elevations of glucocorticoid levels can directly decrease testosterone levels without altering LH levels [59–61]. Further, in case of chronic stress, a decrease in LH and GnRH levels becomes apparent [62, 63].

#### Heat stress on gonads

In males, testes are suspended in a scrotum outside the body in order to keep the temperature 2 to 4 °C lower than that of core body temperature. This is a requirement for normal spermatogenesis [64]. However, heat stress to the testes not only decreases semen quality but also indirectly lowers embryo quality after fertilization as the spermatozoa produced in overheated testis exhibits damage [65–67]. In this context, heat stress is responsible for enhancing ROS production as well as decreasing antioxidant enzyme activities, increasing NADPH oxidase activity and disrupting mitochondrial homeostasis [68, 69]. Numerous reports have documented that factors such as fever, sauna or steam room use, sleeping posture, long time sitting or driving, polyester-lined athletic supports, using a laptop on the lap and electric blankets impose negative effects on scrotal temperatures and subsequently spermatogenesis [70, 71]. Studies have also reported that clinical conditions such as cryptorchidism, varicocele, and acute febrile illness can increase testicular temperature and suppress spermatogenesis [70].

Activation of the hypothalamic–pituitary–adrenal (HPA) axis and the consequent increase in plasma glucocorticoid concentrations are two of the most important responses to heat stress. Heat stress imparts detrimental effects on male reproduction partly by disrupting the normal release of GnRH from the hypothalamus as well as LH and FSH from the anterior pituitary gland [72]. Several studies have indicated that testicular heat stress leads to a decline in the circulating levels of testosterone and LH but increases serum cortisol levels [73, 74]. Testicular heat stress also leads to Leydig cell apoptosis and a reduction in testosterone biosynthesis in adult rat testes [75]. Moreover, increased testicular temperature adversely affects Sertoli cell function, production of testicular androgen-binding protein, spermatogenesis and semen parameters [76]. Thus, increased heat stress elevates the generation of ROS in the male reproductive tract by directly affecting cellular metabolism [69] and by influencing stress hormone levels [77]. The resulting increase in ROS production, in turn, damages testicular germ cells and other endocrine cells to disrupt the hormonal balance, thereby curbing male fertility [34].

#### Environmental toxicants

Exposure to environmental contaminants adversely affects the male reproductive potential [78, 79]. Male infertility caused by exposure to environmental toxicants such as cadmium [80, 81], mercury [82, 83], bisphenol A (BPA) [84, 85] and dioxin [86] is a worldwide problem. Even chemical components of air pollution can induce OS by triggering redox-sensitive pathways subsequently leading to various malaise, such as inflammation and cell death [87].

These contaminations deteriorate semen parameters, DNA integrity via disrupting Leydig and Sertoli cell function, hormone biosynthesis, gene expression and epigenetic modifications [12, 88, 89]. These toxicants commonly act as ‘endocrine disrupting chemicals’ (EDCs) that interfere with normal hormonal functions [90], enhance the level of circulating cortisol owing to OS induction [91] and reduces circulating testosterone levels [92, 93]. Increased cortisol decreases LH secretion through crosstalk between the HPG-HPA axes. Decreased LH concentration fails to stimulate the Leydig cells resulting in decreased testosterone production, whereas decreased FSH affects normal Sertoli cell functions [94]. These toxicants also interfere with the cellular communications and adhesions between Sertoli–Sertoli cells and Sertoli–germ cells via the phosphatidylinositol 3-kinase (PI3K)/c-Src/focal adhesion kinase (FAK) signalling pathway which leads to reproductive dysfunction [95] and disrupted hormonal secretion. Thus, these toxicants disrupt normal male reproductive hormonal balance by their disruptive influence upon the endocrine and reproductive organs as well as by interfering in the cross-talk among different endocrine axes [96].

#### Electromagnetic radiations

Since the last few decades, it has been widely reported that long-term exposure to electromagnetic radiations can generate ROS in reproductive organs, which not only declines motility, viability, and normal morphology of functional spermatozoa [97, 98], but also disorients reproductive hormonal profiles. The use of cell phones [99], wireless internet [100] and other occupational or environmental radiations [101] are found to be major causative factors directly augmenting ROS generation in male reproductive organs [102, 103]. Electromagnetic radiation affects the HPA axis and increases adrenocorticotropic hormone (ACTH) secretion from the anterior pituitary thereby increasing the production of cortisol from adrenal cortex [104]. These radiations can also decrease testosterone secretion from Leydig cells by disrupting the male reproductive hormonal axis [105]. Electromagnetic radiation significantly affect LH levels but not FSH and PRL levels [106]. It has also been reported that exposure to electromagnetic waves directly affects the pineal gland, thereby deteriorating the biological effect of melatonin on GnRH pulse in the hypothalamus [107]. Thus, altered GnRH levels influence FSH and LH secretion and negatively affects testosterone synthesis in the testis [108].

#### Exercise

Contrary to regular exercise that enhances antioxidant defences in the body, unaccustomed and/or exhaustive exercise can lead to the undesirable generation of excessive ROS [109]. Although the exact redox mechanisms remain elusive, it seems that mitochondria, NADPH oxidase (NOX), and xanthine oxidase (XO) are the major endogenous sources of ROS in skeletal muscle [109]. Some studies showed that moderate physical activity can increase FSH, LH, and testosterone levels [110], which is widely associated with increased energy and muscle strength [111, 112]. Despite the impact of moderate exercise, data suggest that vigorous exercise may decrease LH, FSH, and testosterone levels as well as semen parameters [113, 114]. However, other investigators have reported that testosterone levels remain unaltered following heavy exercise [115, 116].

#### Obesity

Obesity is a complex health disorder that severely affects hormonal balance [117]. Obesity disrupts serum levels of leptin [118], ghrelin [119], adiponectin [120], orexin [121], obestatin [122] and other metabolic hormone profiles [117]. Reportedly, leptin correlates positively with body fat mass [123, 124] and a leptin-induced generation of ROS in human endothelial cells result from increased mitochondrial fatty acid oxidation [123, 124]. The activation of the HPG axis could be enhanced by leptin and thus stimulate the release of GnRH, FSH and LH [125]. Moreover, leptin can directly affect the gonads due to its receptor isoforms in gonadal tissue [125].

Though the impact of ghrelin on serum testosterone level is contentious [126–128], it is reported that ghrelin receptors are present in the testis and that ghrelin plays a key role in testosterone production, but not directly in spermatogenesis [126]. Increased ROS levels appear to cause increased levels of ghrelin [129] which may, in turn, result in obesity and further ROS production.

Serum adiponectin level is negatively correlated with both testosterone [130] and ROS production [131]. Orexin (hypocretin) is known to stimulate testosterone production by enhancing the activities of steroidogenic enzymes in Leydig cells [132]. It is also reported to attenuate ROS-induced cell damage [133]. All these metabolic hormones either directly or indirectly reduce the androgen profile in men.

The complex cross-talk among these hormones is interrupted in obesity, thus causing a massive annihilation of the hormonal milieu, which in turn affects male reproductive functions. Although there is a body of evidence highlighting the complexity and the multifactorial effects that obesity has on certain male reproductive functions, the correlation between obesity and semen parameters is still debated [134, 135].

#### Food intake

There is an inverse relationship between the dietary intake of antioxidant-rich food and incidence of human diseases [136]. Many naturally-occurring antioxidant compounds from plant sources have been identified as free radicals or active oxygen scavengers [136]. Studies show that men who consume high dietary fish, fruits, vegetables, legumes, whole grains and omega-3- and omega-6-fatty acids have better semen parameters compared with men consuming high fat, caffeine (> 800 mg/day), red meat, processed meat, pizza, sugary drinks, and sweets in their diet [137, 138]. Therefore, in order to compensate for poor nutritional vitamin intake, food and medicine are routinely supplemented with synthetic and natural food antioxidants.

It is well-known that chronic high-fat and high-protein diets lead to an increase in ROS generation and subsequently OS [139, 140] by disrupting the antioxidant defence [140] and mitochondrial metabolism [139, 141]. This in turn negatively impacts semen quality through alteration of hormone levels [142, 143]. Antioxidant therapies may possibly have a beneficial impact on semen parameters, probably by protecting semen from ROS, reducing OS and improving basic sperm parameters. This improvement can be established by stimulation of testosterone biosynthesis, FSH and LH secretion, inhibin B and enhancement of androgen profile [144]. Investigators have showed that mainly selenium, coenzyme Q10 (CoQ10), and N-acetyl-cysteine can affect semen parameters by increasing testosterone and inhibin B [145]. However, further research is warranted to determine if there are any appropriate antioxidant compounds as well as suitable doses that could potentially be used in clinical practice.

#### Alcohol

Alcohol consumption promotes the generation of ROS through its metabolism pathway in the liver by stimulating the activity of cytochrome P450 enzymes, alteration of certain levels of metals (particularly free iron or copper ions) in the body, and finally, reduction in the antioxidant levels [146]. Due to the critical contribution of certain metals (particularly iron and copper) to the production of hydroxyl radical, anything that increases the levels of these metals can also promote ROS generation and OS [147]. It has been reported that alcohol increases iron levels in the body not only by iron-rich alcoholic beverages, such as red wine, but also by enhancing the absorption of iron from food [148].

Evidences in both animals and humans show that alcohol is also associated with high levels of estradiol and this finds relevance in the fact that estradiol enhances beta-endorphin release that is conventionally linked with the effects of alcohol consumption [149]. Chronic alcohol consumption can reduce serum testosterone, LH, and FSH levels by affecting the interactions between the neural and endocrine systems [149, 150]. Alcohol disrupts the cleavage of GnRH molecule from its precursor pre-pro GnRH and prevents the movement of protein kinase C15 which is necessary for the GnRH-stimulation of LH and FSH [151, 152]. Eventually, this disrupts the endocrine balance and subsequently affects semen parameters [153].

Among testicular cells, Sertoli cells are those that are most affected by chronic alcohol consumption [154]. Since Sertoli cells contribute the most to testicular size, chronic alcohol abuse eventually causes testicular atrophy, degeneration of germ cells, decreased size of lumen of seminiferous tubules, an abundance of lipid droplets, vacuoles, dilatation of the blood vessels, variation in seminal vesicle diameter as well as apoptosis of Sertoli cells. Due to the intratesticular cross-talk between Sertoli and Leydig cells, Leydig cells are eventually also affected by these changes [154, 155]. Though the correlation between alcohol consumption and infertility seems to be dose-dependent, the threshold of alcohol consumption beyond which would affect male fertility remains ambiguous [156].

#### Opioids, narcotics and recreational drugs

Opioids administration is associated with disrupted spermatogenesis and reduced sexual performance [157]. Both endogenous and exogenous opioids inhibit GnRH secretion, by disrupting the functions of HPG axis [158]. They reportedly generate ROS [159], induce inflammation as well as aid DNA/chromosomal damages and apoptosis in cells by p53 [160, 161]. Opioid consumption leads to increase in serum concentrations of sex hormone binding globulin (SHBG), a protein which tightly binds testosterone and E2 thus restricting the levels of unbound testosterone [162, 163]. Therefore, for opioid users, the level of total testosterone and E2 remain subnormal [162, 163]. Consequently, decreased testosterone levels also result in the decrease of LH levels. The loss of integrity of the HPG axis via opioid actions on sex hormones and LH levels, lead to clinical hypogonadism [162, 164]. The opioid methadone is also reported to significantly reduce testosterone levels by directly affecting steroidogenesis [158].

Marijuana contains the cannabinoid, delta-9-tetrahydrocannabinol (THC), which inhibits GnRH release and LH production [164]. Thus, THC, by imposing adversities upon the HPG axis and causing dose-dependent reduction in testosterone production, impairs spermatogenesis [164, 165] at different mitotic and meiotic stages, resulting in several morphogenetic sperm defects as well as gynecomastia, impaired libido, erectile and ejaculatory dysfunction [166].

Studies showed that heroin can decrease gonadotropin and testosterone levels by affecting the HPG axis [158]. Similarly, cocaine exposure can also disrupt normal gonadal functions and are associated with decreased testosterone production and HPG axis dysregulation [167].

Non-medical use of drug narcotics, such as hydrocodone and oxycodone can interfere with spermatogenesis through their effects on the hypothalamus, and suppress LH release [164].

#### Smoking

Smoking is a well-known cause of male subfertility/infertility [168]. A major mechanism for this effect appears to be ROS production by the interference of oxygen delivery to the testis which compromises the high metabolic requirements of spermatogenesis [168–170]. Smoking also releases a large number of mutagens and metabolites (including radioactive polonium, cadmium, benzopyrene, carbon monoxide, tar, naphthalene, and aromatic hydrocarbons) which disrupt the normal structure and function of the male reproductive organs [168, 169]. It may enhance OS not only directly through the production of reactive oxygen radicals in cigarette smoke, but also indirectly through the weakening of the antioxidant defence systems [171–173]. Studies have indicated that exposure to smoke can change plasma levels of testosterone, PRL, E2, FSH, LH and SHBG by effects on Leydig and Sertoli cells [171–173]. Studies have also shown that smoking is associated with alterations in semen quality of both fertile and infertile men by affecting pituitary, thyroid, adrenal and testicular functions [174].

#### Anabolic steroids

Regular consumption of exogenous steroids can produce ROS by disrupting mitochondrial respiratory chain complexes and lead to the release of inflammatory cytokines and apoptosis [175]. Exogenous steroid hormones inhibit spermatogenesis by suppressing the HPG axis, thus limiting the release of FSH and LH and in turn decreasing testosterone biosynthesis in the testis [176, 177]. Hypogonadism associated with anabolic androgenic steroid (AAS) abuse is usually reversible within 3–6 months after discontinuation. However, complete recovery takes more than 3 years or may even be impossible to achieve [164]. AAS abuse primarily produces Leydig cell alterations which lead to a decrease in testosterone synthesis [177]. However, disruption in the end stage of spermatogenesis with a lack of mature spermatozoa (oligozoospermia/ azoospermia), testicular atrophy, and morphologically-abnormal sperm have been reported in AAS consumers [178]. Following AAS discontinuation, Leydig cells start further proliferation but cellular counts generally remain less than normal, accounting for delayed recovery of testosterone levels and the occasional irreversible effects of AAS [179].

### Endogenous factors

Though endogenous ROS is necessary for normal male reproductive functions, its excessive production may interfere with the endocrine axes and their cross-talk.

#### Aging

In the aged male, Leydig cells are oxidatively damaged due to excessive generation of endogenous ROS and decreased concentration and activity of antioxidant enzymes [180]. As a result of excessive ROS generation, oxidative modifications of DNA and alterations in the mitochondrial membrane potential required for testosterone synthesis take place [181, 182]. Alongside these changes, an increase in LH sensitivity due to diminishing LH receptors per cell and a reduced ability of LH to activate steroidogenic acute regulatory (StAR) protein, which transport cholesterol from the outer mitochondrial membrane to the inner, occurs [183, 184]. Thus, overproduction of ROS may play a role in age-related testicular degeneration associated with male infertility [185].

The steroidogenic steps regulated by the P450 enzymes are the most likely sites of ROS action [186, 187]. FSH and human chorionic gonadotropin (hCG) together have been reported to stimulate ROS-producing cellular metabolisms affecting differentiation processes in germ cells [185, 188, 189]. Furthermore, following ROS production, the activities of several enzymes of the testosterone biosynthetic pathway are reduced, resulting in further decrease in testosterone synthesis and secretion [190, 191].

#### Reproductive tract infections

Reproductive tract infections is an important cause of disrupted male reproductive function and infertility [47]. Many immunoregulatory and pro-inflammatory cytokines are produced by testicular spermatogenic and somatic cells, both under normal conditions as well as during an inflammatory scenario [192]. Cytokines (such as IL–1, IL–6 or TNF-α) are even produced by non-immune cells like Leydig cells and Sertoli cells, that appear as typical components of seminal plasma to maintain normal spermatogenesis [192, 193]. Reproductive tract infections can be caused by ejaculatory duct inflammation, epididymitis, sexually transmitted infections (e.g. gonorrhoea, *Chlamydia trachomatis*, *Escherichia coli*, mycobacteria and *Ureaplasma urealyticum*), urethritis, testicular torsion, varicocele and several other causes like chronic prostatitis, inflammation of one or both testes (orchitis), and even by some drug therapy (escitalopram, tramadol, levonorgestrel etc.) [47, 194]. With the progression of inflammatory damage and weakening of antioxidant defence, as a mitigation strategy against the colonised bacterial strains, there can be increased ROS levels in the male genital tract, affecting the prostate gland, seminal vesicles or the epididymis [47, 195].

Reproductive tract infections indirectly cause germ cell degeneration and disruption of spermatogenesis through either of the following occurrences [196]: (i) changes in testicular temperature following high fever; (ii) congestion of seminiferous tubule following interstitial oedema; or (iii) modification of testosterone production. Though studies on male sex hormones and reproductive tract infections are scanty, some investigators observed the reduction of testosterone together with an increase in LH and FSH levels in patients with reproductive tract infections [196–198]. It has been reported that in patients with chronic prostatitis, corticosterone level decreases, while testosterone level increases compared to normal controls [199]. Whereas in mumps orchitis, increased corticosterone level decreases both LH and FSH levels which results in reduced production of testosterone from Leydig cells [200].

## Hormonal influence on the oxidative status of male reproduction

OS that occurs due to either the enhanced production of ROS or reduced availability of antioxidants may cause lipid peroxidation in Leydig cells and germ cells, damage to lipoproteins, protein aggregation and fragmentation, and steroidogenic enzyme inhibition [10]. Testicular OS causes a reduction in testosterone production, either as a result of the injury to the Leydig cells or to other endocrine structures like the anterior pituitary [201, 202]. Reportedly, normal steroidogenesis also generates ROS, which are largely produced by mitochondrial respiration and the catalytic reactions of the steroidogenic cytochrome P450 enzymes [186]. ROS generated in this way, in turn, have been identified to inhibit subsequent steroid productions, and to damage mitochondrial membranes of spermatozoa [203]. OS is associated with increased numbers of immature spermatozoa via an indirect effect on the male hormone production that is correlated with spermatogenesis [204, 205].

It has been reported that systemic hormones (FSH, LH, testosterone, E2, PRL) may regulate seminal total antioxidant capacity (TAC) [206, 207]. A positive relationship between PRL or free T4 (fT4) and a negative correlation between gonadotropins or gonadal steroids with TAC have also been shown [22]. It is evident that some hormones like testosterone and MLT may act as antioxidants to protect sperm and other testicular cells from damage induced by ROS [208, 209]. Other metabolites of the steroidogenic pathway like DHEA are reported to enhance the level of cellular antioxidants, but the proper mechanism is still unclear [210]. Direct and indirect relationships between testosterone and antioxidant levels like selenium and/or CoQ10 and between testosterone and zinc in infertile men, respectively, have been observed [207, 211]. CoQ10 can decrease FSH and LH levels [212]. A negative relationship has been found between serum level of testosterone, E2, fT4 and sperm DNA damage [213, 214]. Also, the antioxidant inhibition could affect triiodothyronine (T3), thyroxine (T4), neurotransmitter noradrenaline and increase sperm DNA damage [215]. Intramuscular or subcutaneous injection of highly purified FSH to idiopathic infertile men reduces ROS production [216] and the subsequent sperm DNA damage [217]. Although it has been reported that testosterone could produce DNA fragmentation in Sertoli and germ cells by stimulating caspase activities in Sertoli cells [218], long-term effects of antioxidants can alter FSH, testosterone, and inhibin B levels [219].

## Mechanism of action

Innumerable exogenous and endogenous factors, as discussed above, can produce ROS in the male reproductive system by disrupting the balance of oxidants and antioxidants. Following the generation of ROS, the HPA axis becomes activated and releases corticosterone (in animals) and cortisol (in humans) in response to stress. These stress hormones, through the cross-talk between the HPG and HPA axes, negatively affect LH secretion from the anterior pituitary. Decreased LH fails to stimulate Leydig cells to produce enough testosterone. Decreased FSH diminishes the release of androgen-binding protein (ABP) from the Sertoli cells, and thus, an overall decline in circulating testosterone occurs during severe OS.

ROS also affect HPT axis to reduce T3 and T4 secretion. Decreased T3 reduces the levels of the StAR mRNA and protein in Leydig cells, as well as testosterone production [220]. Increased OS also decreases the secretion of insulin from the pancreas which further negatively affects T3 release from the thyroid gland and thereby testosterone biosynthesis.

Conditions such as obesity not only involve the HPA and HPT axes, it also includes several metabolic hormones that manifest ROS-induced alterations in male reproductive functions. Obesity-induced ROS can affect adipocytes to secrete more leptin, which together with insulin, negatively regulate T3-release and thereby inhibit testicular functions. Leptin, secreted by adipocytes also inhibit GnRH release from the hypothalamus.

Testicular E2 and inhibin are produced intensely during OS, which then inhibit testosterone release. Following ROS exposure, aromatase activity increases which result in more E2 production. ROS exposure is also reported to increase PRL secretion from anterior pituitary which causes decreased GnRH release. Infections in the reproductive tract can lead to the production of pro-inflammatory cytokines (TNF-α, IL-1b, and IL-6) which again inhibit both GnRH release and testosterone secretion.

Thus, through its actions on an individual hormonal axis and/or by disrupting the cross-talk among different endocrine systems, ROS can lead to decreased testosterone production as the outcome of endocrine disruption. Decreased testosterone fails to regulate spermatogenesis properly to produce enough mature spermatozoa. It also fails to maintain the normal growth of accessory reproductive organs which play crucial roles in sperm maturation. As a prime regulator of male reproductive behaviour, testosterone deficiency may lead to suppressed sexual behaviour among men. Thus, by disrupting the endocrine reproductive functions, ROS may result in male infertility (Fig. 1).

## Conclusion

This review summarizes the alterations of the reproductive endocrinological status by numerous endogenous and exogenous sources of ROS. Pivotal hormonal regulators of male reproductive functions can be affected by the disruption of the balance between ROS production and the antioxidant defence mechanism in the male reproductive system. Uncontrolled generation of ROS may directly damage reproductive tissues or can interfere with the normal regulatory mechanisms of the HPG axis and its crosstalk with other endocrine axes, to adversely affect male reproductive functioning, thereby inducing male infertility.

## Abbreviations

11β-HSD: 11β-hydroxysteroid dehydrogenase
AAS: Anabolic androgenic steroid
AMH: Anti-Mullerian hormone
CORT: Corticosterone
delta-9-THC: Delta-9-tetrahydrocannabinol
DHEA: Dehydroepiandrosterone
E2: Estradiol
FSH: Follicle-stimulating hormone
fT4: Free T4
GC: Glucocorticoid
GnRH: Gonadotropin releasing hormone
HPG: Hypothalamic-pituitary-gonadal
LH: Luteinizing hormone
MLT: Melatonin
NOX: NADPH oxidase
OS: Oxidative stress
PRL: Prolactin
ROS: Reactive oxygen species
SHBG: Sex hormone binding globulin
TAC: Total antioxidant capacity
XO: Xanthine oxidase
